# DNA-PK Inhibitor, M3814, as a New Combination Partner of Mylotarg in the Treatment of Acute Myeloid Leukemia

**DOI:** 10.3389/fonc.2020.00127

**Published:** 2020-02-13

**Authors:** Michael I. Carr, Astrid Zimmermann, Li-Ya Chiu, Frank T. Zenke, Andree Blaukat, Lyubomir T. Vassilev

**Affiliations:** ^1^Translational Innovation Platform Oncology, EMD Serono Research and Development Institute, Inc., Billerica, MA, United States; ^2^Translational Innovation Platform Oncology, Merck KGaA, Darmstadt, Germany

**Keywords:** DNA-PK, ADC-antibody drug conjugate, AML-acute myeloid leukemia, therapy, DSB-double-strand break

## Abstract

Despite significant advances in the treatment of acute myeloid leukemia (AML) the long-term prognosis remains relatively poor and there is an urgent need for improved therapies with increased potency and tumor selectivity. Mylotarg is the first AML-targeting drug from a new generation of antibody drug conjugate (ADC) therapies aiming at the acute leukemia cell compartment with increased specificity. This agent targets leukemia cells for apoptosis with a cytotoxic payload, calicheamicin, carried by a CD33-specific antibody. Calicheamicin induces DNA double strand breaks (DSB) which, if left unrepaired, lead to cell cycle arrest and apoptosis in cancer cells. However, repair of DSB by the non-homologous end joining pathway driven by DNA-dependent protein kinase (DNA-PK) can reduce the efficacy of calicheamicin. M3814 is a novel, potent and selective inhibitor of DNA-PK. This compound effectively blocks DSB repair, strongly potentiates the antitumor activity of ionizing radiation and DSB-inducing chemotherapeutics and is currently under clinical investigation. Suppressing DSB repair with M3814 synergistically enhanced the apoptotic activity of calicheamicin in cultured AML cells. Combination of M3814 with Mylotarg in two AML xenograft models, MV4-11 and HL-60, demonstrated increased efficacy and significantly improved survival benefit without elevated body weight loss. Our results support a new application for pharmacological DNA-PK inhibitors as enhancers of Mylotarg and a potential new combination treatment option for AML patients.

## Introduction

It is estimated that over 20,000 people in the U.S. will be diagnosed with AML in 2019 (1). Despite established standards of induction and consolidation therapies, the overall 5 years survival rate is ~30%, and U.S. statistics show few changes in per capita AML deaths in the last two decades (1). Strong chemotherapeutic regimens remain the standard approaches to AML treatment, and patient mortality is often linked to treatment-related toxicities or ablated normal hematopoiesis (2). Further limitations arise when considering the advanced median age of diagnosis (68 years), and associated health-liabilities, when facing broadly cytotoxic treatments. Accordingly, death rates from AML are higher in patients over 65 years of age (1). Therefore, there is a clear need for novel, targeted therapeutic strategies for AML. Such therapies would ideally display high potency toward the leukemic burden and improved tolerability in normal tissues.

Antibody drug conjugates (ADCs) are targeted therapeutics with broad potential for anti-cancer efficacy through their diverse target antigens and drug payloads. ADCs consist of recombinant monoclonal antibodies (mAbs) that are covalently bound to cytotoxic chemical agents (commonly referred to as warheads) via synthetic linkers. These conjugates facilitate the delivery of highly cytotoxic small-molecule drugs with the high selectivity, stability and pharmacokinetic profile of mAbs (3). The first ADC to receive FDA approval, gemtuzumab ozogamicin (Mylotarg^TM^), leverages the selectivity of an anti-CD33 antibody to target AML cells. The transmembrane glycoprotein CD33 is expressed on the surface of leukemia blasts in most patients with AML, but not on normal hematopoietic stem cells (4). Mylotarg consists of a humanized IgG_4_ anti-CD33 antibody conjugated to the calicheamicin derivative N-acetyl-γ-calicheamicin dimethyl hydrazide (4). Its anti-leukemic activity is derived from the internalization and lysosomal localization of CD33/Mylotarg complexes, wherein the acidic environment facilitates linker hydrolysis and release of the cytotoxic calicheamicin moiety (4–6).

Calicheamicin is a member of the enediyne family of antibiotics, which binds the minor groove of DNA in a sequence-specific manner and is reduced to form a reactive intermediate that triggers the formation of DNA double strand breaks (DSB) and single strand breaks (SSB) (7–10). Calicheamicin-induced DNA damage leads to cell cycle arrest to assist DNA repair machinery or, if damage is unrepairable, apoptosis and cell death (11). DNA repair and/or the associated cell fate decisions occur through activation of the ataxia telangiectasia mutated (ATM), ataxia telangiectasia and rad3-related (ATR) and DNA-dependent protein kinase (DNA-PK)-driven pathways (12, 13). Accordingly, cells defective for ATM or DNA-PK are hypersensitive to calicheamicin (9, 14). Given the apparent impact of DNA-PK activity on cells sensitivity to calicheamicin-induced DNA damage, we hypothesized that pharmacological inhibition of DNA-PK catalytic activity may enhance the anti-leukemic effects of Mylotarg by potentiation of the cytotoxicity of its warhead, calicheamicin. M3814 belongs to a new generation of potent and selective inhibitors of DNA-PK protein kinase (15, 16). It effectively blocks DSB repair driven by DNA-PK via the non-homologous end joining (NHEJ) mechanism and strongly potentiates the antitumor activity of ionizing radiation and DSB-inducing chemotherapy agents (15). M3814 has completed Phase 1 evaluation as a single agent and currently undergoing clinical investigation in combination with radiotherapy and/or immunotherapy in solid tumors.

The guardian of the genome, p53, is a central regulatory node in DNA damage response shown to be a key player in protecting or destroying cells with damaged DNA to suppress tumor formation (17, 18). Recently, we reported that p53 plays a critical role in determining cell fate in the response of irradiated cancer cells to DNA-PK inhibitor, M3814 (16). Blocking DSB repair in cancer cells expressing wild-type p53 causes overactivation of the ATM signaling axis with a substantial boost of p53 activity and reinforcement of ATM/p53-controlled cell cycle checkpoints, leading to a complete cell cycle arrest and premature cell senescence. Cancer cells with dysfunctional p53 were not protected from entry into mitosis with unrepaired DSBs, causing severe chromosomal aberrations, mitotic errors and apoptotic cell death. This new mechanistic model for response to combined treatment with DSB-inducing agents and a DNA-PK inhibitor was derived from solid tumor cell lines which have been shown to retain their p53-dependent cell cycle arrest but frequently lose their p53 apoptotic activity (19). Acute leukemia cells, however, are known to preserve their ability to undergo effective apoptosis and cell death in response to p53 pathway activation (20). Therefore, the M3814-induced p53 activity boost in response to DSBs could further potentiate the activity of calicheamicin in AML cells, the majority of which express wild-type and functional p53. Here, we show that suppressing DSB repair with M3814 synergistically enhances the anti-leukemic activity of calicheamicin in cultured AML cells through p53 dependent and independent mechanisms. Combination of the selective DNA-PK inhibitor with Mylotarg in two AML xenograft models is well-tolerated, provides significant efficacy and survival benefit compared to Mylotarg alone and could offer a new combination approach to AML therapy.

## Materials and Methods

### Cell Lines and Reagents

All cell lines were obtained, mycoplasma free, from the Merck Tissue Culture Bank (Merck KGaA, Darmstadt, Germany). Cells were originally purchased from ATCC (Manassas, Virginia), or DSMZ (Braunschweig, Germany) and kept in liquid nitrogen at low passage until use. Cell line identity was confirmed by short tandem repeats (STRs) analyses and mycoplasma infection was excluded by PCR-based testing. Molm-13, MV4-11, and HL-60 cells were maintained in RPMI 1640 media (GIBCO, Gaithersburg, MD) supplemented with 20% heat-inactivated fetal bovine serum (FBS). AML cell lines were cultured at low confluency and no longer than 20 passages to avoid cell differentiation. Culture medium was supplemented with 10% fetal bovine serum (FBS) (Corning Life Science, Tewksbury, MA, USA). M3814 and M3814R were synthesized at Merck KGaA, Darmstadt, Germany. Calicheamicin was purchased from MedChemExpress (Monmouth Junction, NJ, USA). All compounds were dissolved in DMSO to prepare stock solutions and kept frozen at −20°C until use. The final concentration of DMSO in media did not exceed 0.1% (vol/vol). Mylotarg (gentusumab ozogamicin for injection, Pfizer, 5 mg/vial) was purchased from Asaman, Inc. (Avon, MA, USA).

### Cell Growth and Viability Assays

For drug combination matrix cell growth/viability testing, cells were plated at 2 × 10^4^ cells/ml overnight in 96-well plates. The next day, cells were treated with the indicated concentrations of M3814 and calicheamicin. Drugs were added using D300e digital dispenser (Tecan) in DMSO with diluent normalization (DMSO 0.1%). Effect on cell growth/viability was assessed at 48, 72 or 144 h using the CellTiterGlo 2.0 Cell Assay (Promega, Madison, WI, USA) according to manufacturer's protocol. Luminescence was detected using an EnVision plate reader (Perkin Elmer, Waltham, MA, USA). Calicheamicin dose-response curves and IC50 values for fixed M3814 doses were generated by graphing relative viability and curve fitting using GraphPad Prism (v8.0.1). Synergism between M3814 and calicheamicin was calculated and graphed according to Bliss and Loewe models using Combenefit software (v. 2.021) (21). Cell counting assays were performed by plating 2 × 10^5^ cells/well in 6-well plates and treating in triplicate with the indicated drugs. Viable cells were counted at specified timepoints using trypan exclusion and a Cellometer Mini automated cell counter (v1.2.3.3) (Nexcelom Bioscience, Lawrence, MA, USA).

### Live-Cell Imaging

Live-cell imaging was performed using an IncuCyte ZOOM live cell analysis system (Essen BioScience, Ann Arbor, MI, USA). 2,000 cells/well were plated in poly-d-lysine coated, black walled 96-well plates (Becton Dickinson, Bedford, MA, USA). Incucyte Annexin V Green reagent was added to media as per manufacturer's instructions (Essen BioScience, Ann Arbor, MI, USA). Cells were treated with the indicated concentrations of M3814 and calicheamicin and imaged using a 10x objective at 2 h intervals for 5 days. Relative apoptosis was calculated as Annexin V-positive events per mm^2^ normalized to percent confluence.

### Western Blot Analyses

MV4-11 cells plated at 1–5 × 10^5^ cells/ml in T75 flasks were treated with the indicated drugs. Lysates were prepared at 6, 24, 48, or 96 h using RIPA lysis buffer (Cell Signaling Technology, Danvers, MA) supplemented with protease and phosphatase inhibitors (Roche Diagnostics, Indianapolis, IN, USA). Lysates were resolved using NuPAGE 4–12% Bis-Tris, or 3–8% Tris-Acetate gels (Thermo Fisher Scientific, Waltham, MA, USA), and transferred to Nitrocellulose membranes with an iBlot 2 Gel Transfer Device (Thermo Fisher Scientific, Waltham, MA, USA). Membranes were treated and imaged with a LI-COR Odyssey CLx imaging system in accordance with the LI-COR Near-Infrared (NIR) Western Blot Detection Protocol (LI-COR, Lincoln, NE, USA). Primary antibodies were as follows: p-ATM (S1981) (#ab81292, Abcam Biotechnology, Cambridge, MA, USA); ATM (#MA1-23152, Thermo Fisher Scientific, Waltham, MA, USA); p-CHK2 (T68) (#2197, Cell Signaling Technology, Danvers, MA, USA); CHK2 (#6334, Cell Signaling Technology, Danvers, MA, USA); p-p53 (S15) (#9284, Cell Signaling Technology, Danvers, MA, USA); p53 (#48818, Cell Signaling Technology, Danvers, MA, USA); p21 (#2947, Cell Signaling Technology, Danvers, MA, USA); MDM2 (#sc-965, Santa Cruz Biotechnology, Dallas, TX, USA); Puma (#12450, Cell Signaling Technology, Danvers, MA, USA); Cleaved PARP (#5625, Cell Signaling Technology, Danvers, MA, USA); Cleaved Caspase 3 (#9664, Cell Signaling Technology, Danvers, MA, USA), and Vinculin (#V9131, Sigma-Aldrich, St. Louis, MO, USA).

### Gene Expression Analysis

RNA was isolated using RNeasy Mini Kit with on column DNase digestion (Qiagen, Germantown, MD, USA). RNA purity and concentration were determined using a Nanodrop spectrophotometer (Thermo Fisher Scientific). cDNA was synthesized using Superscript IV Vilo Master Mix (Thermo Fisher Scientific) as described by manufacturer. Quantitative PCR was performed using TaqMan Fast Advanced Master Mix and a 7500 Fast Dx Real-Time PCR Instrument (Applied Biosystems). TaqMan probes used were CDKN1A (Hs00355782_m1), MDM2 (Hs01066930_m1), BBC3 (Hs00248075_m1) and GAPDH (Hs02758991_g1). Relative fold-change gene expression was normalized to GAPDH.

### Cell Cycle and Apoptosis

For cell cycle and apoptosis analyses, 0.4–2 × 10^6^ cells/well were plated in 6-well plates and treated in triplicate with the indicated drugs. At specified timepoints, 1 × 10^6^ cells were either fixed in 70% ethanol and stained with 7-AAD (7-amino-actinomycin D) (BD Biosciences, San Jose, CA, USA) for cell cycle analysis, or stained with phycoerythrin (PE) conjugated Annexin V and 7-AAD for apoptosis analysis, using the PE Annexin V Apoptosis Detection Kit I (BD Biosciences, San Jose, CA, USA). Samples were analyzed using a BD FACSCanto flow cytometer (BD Bioscience, San Jose, CA) and data was processed using FlowJo software (v10.6.1) (FlowJo, LLC).

### Animal Studies

Study designs and animal usage were approved by local animal welfare authorities (Regierungspräsidium Darmstadt, Hesse, Germany). For MV4-11 xenograft studies, female, 8–10 weeks old H2d Rag2 [C;129P2-H2^d^-TgH(II2rg)^tm1Brn^-TgH(Rag2)^tm1Alt^N4] mice (Taconic Biosciences, Denmark) were used. 2.5 × 10^6^ tumor cells were injected subcutaneously in the flank, in 100 μl of 1:1 (v:v) Dulbecco's phosphate-buffered saline (calcium- and magnesium-free)/ Matrigel^TM^ Basement Membrane Matrix (BD Biosciences). Tumors were left to reach 65–180 mm^3^ and mice were randomized into groups of equal mean tumor volume (170 mm^3^) prior to treatment. For HL-60 xenograft studies, female, 6–8 weeks old Hsd:Athymic Nude-Foxn1^nu^ mice (Envigo, France) were used. 2 × 10^6^ tumor cells were injected subcutaneously in the flank, in 100 μl of 1:1 (v:v) Dulbecco's phosphate-buffered saline (calcium and magnesium free)/Matrigel^TM^ Basement Membrane Matrix (BD Biosciences). Tumors were left to reach 94–284 mm^3^ and mice were randomized into groups of equal mean tumor volume (170 mm^3^) prior to treatment. M3814 was suspended for oral administration in a vehicle of 0.5% Methocel/0.25% Tween20 in 300 mM citrate buffer, pH 2.5. Mylotarg was formulated for intravenous administration according to the package insert by reconstituting the lyophilizate to a concentration of 1 mg/ml in water for injection and diluting to final concentration using 0.9% saline.

### Statistical Analyses

All statistical tests were performed with GraphPad PRISM version 7.0 (GraphPad Software Inc.). The data were analyzed with Student *t*-tests. *P* ≤ 0.05 were considered statistically significant. All assays were conducted independently three times, unless indicated otherwise, and representative data is shown as mean ± SD. Significance values are ^*^p < 0.05, ^**^p < 0.01, and ^***^p < 0.001. NS stands for non-significant (*p* > 0.05).

## Results

### M3814 Potentiates the Antitumor Activity of Calicheamicin in AML Cells

We have previously shown that the DNA-PK inhibitor M3814 can effectively enhance the antitumor effect of ionizing radiation (IR) by inhibiting NHEJ repair of IR-induced DSBs in solid tumor cells (15, 16). In cancer cells expressing wild-type p53, this effect is largely due to overactivation of the ATM/p53 signaling axis boosting p53 to levels much higher than the levels induced by radiation alone. This is leading to a complete cell cycle arrest and premature cell senescence but not apoptosis (16). We hypothesized that p53 wild-type acute leukemia cells, known to be highly sensitive to p53-induced apoptosis (22), will be more effectively killed by the M3814 mediated p53 boost in response to calicheamicin-induced DSBs.

**Figure 1:**
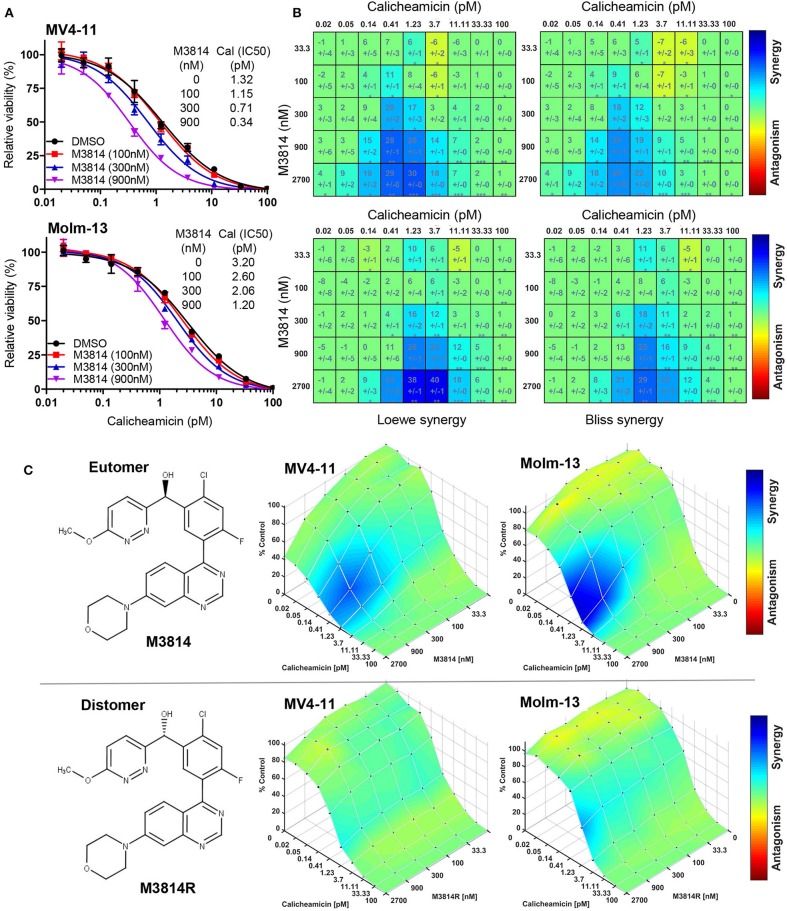
M3814 synergizes with calicheamicin in killing AML cells. **(A)** Proliferating MV4-11 and Molm-13 cells were exposed to increasing concentrations of calicheamicin alone or in combination with three fixed concentrations of M3814 and their viability was measured after 48 h by CellTiter-Glo assay. Relative viability was graphed for fixed M3814 doses and curve fitting performed to generate IC50 values. **(B)** Loewe and Bliss synergy score matrices for MV4-11 and Molm-13 cells treated with combinations of calicheamicin and M3814. Relative viability by CellTiter-Glo assay was processed using Combenefit software to generate synergy score matrices. Boxes/cells include synergy scores above standard deviations and significance indicators. *P < 0.05, **P < 0.01, ***P < 0.001. **(C)** The structure of the pharmacologically active enantiomer (eutomer) M3814 and overlays of Bliss synergy matrices on combination dose response surfaces for MV4-11 and Molm-13 cells treated with calicheamicin and M3814 for 48 h (top). The structure of the pharmacologically inactive enantiomer (distomer) M3814R and overlays of Bliss synergy matrices on combination dose response surfaces for MV4-11 and Molm-13 cells treated with calicheamicin and M3814R for 48 h (bottom). Results were analyzed and graphed using Combenefit software.

To this aim, we first examined whether M3814 potentiates the cytotoxicity of calicheamicin in p53 wild-type AML cells *in vitro*. Exponentially proliferating MV4-11 and Molm-13 cells (both expressing wild-type p53) were exposed to concentration ranges of calicheamicin and M3814, alone and in combination, and the effect on cell growth/viability was determined after 48 h by the CellTiter-Glo assay. Cell viability was reduced across a range of calicheamicin concentrations when combined with increasing doses of M3814 (100, 300, and 900 nM). These concentrations are within the previously defined selectivity range (16). The curve shift and the corresponding IC50 values suggested a combination effect (Figure 1A). Analysis of cell viability across the full range of calicheamicin and M3814 concentrations by two different methods (Loewe and Bliss excess) using Combenefit software (21) revealed similar regions of synergy in the dose matrices for both cell lines (Figure 1B). The concentration ranges at which synergy was observed at this timepoint was 0.2–4 pM calicheamicin for MV4-11 and 0.4–10 pM for the Molm-13 cells. To confirm that the observed synergism between M3814 and calicheamicin is indeed due to inhibition of DNA-PK catalytic activity, we tested the effect of the DNA-PK inhibitor on cell growth/viability of MV4-11 and Molm-13 cells using the pharmacologically active M3814 eutomer (Figure 1C, upper panel) and its distomer, M3814R (Figure 1C, lower panel), shown to be over 20-fold less potent in inhibiting DNA-PK enzymatic activity (16). As expected, synergy was observed in both cell lines exposed to the combination of M3814 and calicheamicin but not in cells treated with M3814R and calicheamicin, indicating that the inhibition of DNA-PK kinase activity underlies the observed synergistic relationship (Figure 1C).

### M3814 Overactivates p53 in Response to Calicheamicin in AML Cells

**Figure 2:**
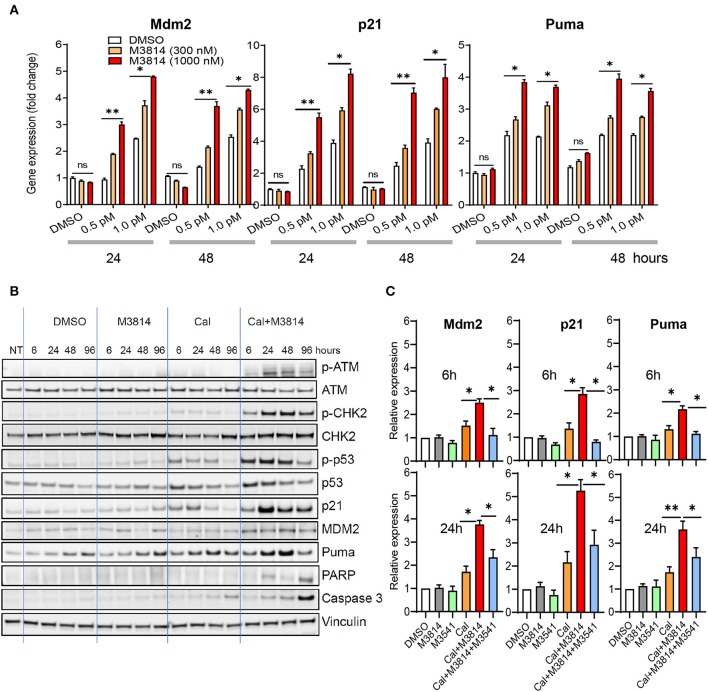
M3814 overactivates p53 in response to calicheamicin in AML cells. **(A)** Relative gene expression analysis of key p53 transcriptional targets, Mdm2, p21 and Puma, in MV4-11 (p53 wild-type) cells treated with DMSO, calicheamicin (0.5 or 1.0 pM), or M3814 (300 or 1,000 nM) alone or in combination. Relative expression determined by the 2^(−ΔΔCt)^ method with GAPDH reference. **(B)** Western blot analysis of ATM and p53 pathway proteins as well as apoptotic indicators at 6, 24, 48, and 96 h in lysates of MV4-11 cells treated with vehicle, M3814 (1 μM), calicheamicin (1pM), or the combination of calicheamicin (1 pM), and M3814 (1 μM). **(C)** Relative gene expression analysis at 6 and 24 h of key p53 transcriptional targets, Mdm2, p21, and Puma, in MV4-11 (p53 wild-type) cells treated with DMSO, M3814 (1 μM), M3541 (1 μM), calicheamicin (1.0 pM), calicheamicin (1 pM) + M3814 (1 μM), or calicheamicin (1 pM) + M3814 (1 μM) + M3541 (1 μM). *P < 0.05, **P < 0.01, ***P < 0.001.

We investigated the effect of the combination treatment with calicheamicin and M3814 on p53 activity in the p53 wild-type MV4-11 cell line. Cells were treated with solvent (DMSO) or calicheamicin (0.5 or 1 pM) and M3814 (300 or 1,000 nM) alone and in combination. These M3814 concentrations were shown to be within the activity range (over 80% DNA-PK inhibition) in most tested cancer cell lines, while remaining selective to its target (16). Gene expression analysis of three key p53 transcriptional targets, responsible for p53 protein stability (Mdm2), p53-dependent cell cycle arrest (p21) and p53-dependent apoptosis induction (Puma), showed a dose-dependent upregulation in response to calicheamicin after 24 and 48 h exposure to the indicated concentrations of single agents or drug combinations (Figure 2A). While M3814 treatment did not affect p53 target gene expression in the absence of calicheamicin-induced DNA damage, combined M3814 and calicheamicin treatment resulted in a dose-dependent 2- to 5-fold increase in expression (Figure 2A). These results indicated that the combination treatment enhances p53 pathway activation in the response to calicheamicin in agreement with our findings in solid tumor cellular models (16).

We then examined the status of ATM/p53 signaling in MV4-11 cells. Exponentially growing cells were exposed to the solvent (DMSO), calicheamicin (1 pM), M3814 (1 μM), and their combination (1 μM M3814 plus 1 pM calicheamicin) and the levels of key proteins from the ATM (p-ATM, p-CHK2) and p53 pathways (p-p53^Ser15^, p21, Mdm2, Puma) were analyzed by Western blotting at 6, 24, 48, and 96 h (Figure 2B). At a concentration of 1 pM, calicheamicin increased slightly the levels of p-ATM^Ser1981^ and its direct phosphorylation target p-CHK2^Thr68^, most notably between 6 and 48 h. The effect on p53 signaling was more pronounced with p-p53^Ser15^ and total p53 levels following the pattern of p-CHK2 activation. The p53 transcriptional targets, p21, Mdm2, and Puma, all had elevated, if slightly different patterns. Cell cycle checkpoint protein p21 was upregulated quickly but its level decreased after 24 h while the apoptosis regulator Puma reached highest levels at 48 h and remained elevated until 96 h post treatment. Mdm2 was elevated more uniformly within the studied period. As seen previously in epithelial cancer cells (16), DNA-PK inhibitor did not show significant effect on all tested proteins except for Puma levels that were slightly higher than the levels in the DMSO control also found to be somewhat elevated at 48 and 96 h. These increases in the control treatments are likely caused by apoptosis in a small fraction of cells due to population overgrowth. The combination of calicheamicin and M3814 induced the expression levels of all tested proteins compared to calicheamicin or M3814 alone at as early as 6 h and caused a pronounced upregulation at 24 h, when p-ATM^Ser1981^ was most elevated. The cell cycle inhibitor, p21, was highest at 24 h and remained elevated until 96 h while calicheamicin-induced p21 was down to basal levels at 96 h. Mdm2 levels were more uniform and higher than in the treatment with calicheamicin alone. The apoptosis inducer, Puma, was also elevated compared to calicheamicin alone and strongest at 48 h. Two markers of apoptosis, cleaved Caspase 3 and cleaved PARP, were also tested. Cleaved caspase 3 was mildly elevated by calicheamicin alone, most notably at 96 h, but significantly stronger in the combination treatment peaking at 96 h. Cleaved PARP elevation was only detected in the combination treatment and was highest at 96 h. These Western blot analyses revealed that M3814 can enhance ATM/p53 signaling in the AML cell line MV4-11 in agreement with our findings in epithelial cancer cell lines (16). The overactivation of the ATM and p53 pathways resulted in significantly higher levels of the p53-dependent controllers of cell cycle and apoptosis, p21 and Puma, predicting stronger cell cycle arrest and/or apoptotic activity.

To confirm the role of ATM activation in the elevated p53 response we used the novel, potent and selective inhibitor of ATM catalytic activity, M3541 (23). Adding 1 μM M3541, a concentration previously shown to inhibit over 80% of ATM autophosphorylation in response to ionizing radiation in multiple cancer cell lines (16, 23), to the combination of calicheamicin and M3814 in MV4-11 cells abrogated the enhancing effect of M3814 on p53 targets, p21, Mdm2, and Puma, bringing them close to untreated levels at 6 h and still significantly reduced relative to the calicheamicin/M3814 combination at 24 h (Figure 2C). ATM inhibitors have limitations as a cellular probe for DSB-induced p53 activation because they simultaneously inhibit its main function as a driver of DSB repair via the homologous recombination pathway (24). Continuous ATM inhibition has been shown to suppress the repair of DSBs and potentiate DSB-inducing agents (23) that may lead to a secondary ATM-independent activation of p53 at later time points. This may explain the incomplete inhibition of the p53 response at 24 h. However, the observed suppression of p53 activation in the early stages of the calicheamicin/M3814 combination treatment is in agreement with our data with solid tumor cell lines (16) and support the hypothesis that p53 activity boost is downstream of M3814-induced ATM overactivation.

### ATM/p53 Pathway Boost by M3814 Contributes to the Antitumor Activity of Calicheamicin in MV4-11 Cells

When activated by genotoxic stress, the p53 pathway can exert two major functions, cell cycle arrest and/or apoptosis to aid in the repair of DNA damage or eliminate damaged cells and suppress tumorigenesis (25). Both functions are activated by many genotoxic chemotherapeutics in p53 wild-type cancer cells and contribute to the antitumor activity of these agents (26). The p53-dependent apoptotic response plays a major role in the therapy of AML which is predominantly p53 wild-type (22). We assessed if the enhanced activation of the p53 pathway by M3814 contributes to potentiation of calicheamicin antitumor activity.

**Figure 3:**
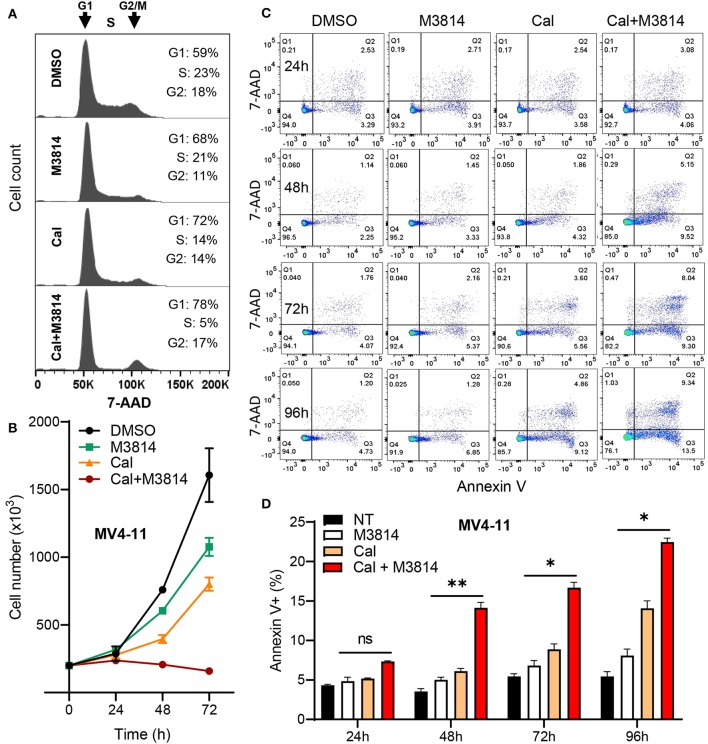
M3814 enhances antitumor activity of calicheamicin in AML cells by a p53-dependent mechanism. **(A)** Cell cycle analysis of 7-AAD stained MV4-11 cells treated with DMSO, M3814 (1 μM), calicheamicin (1 pM), or calicheamicin (1pM) + M3814 (1 μM). **(B)** Cell number as assessed by trypan exclusion at 0, 24, 48, and 72 h for MV4-11 cells treated in triplicate with DMSO, M3814 (1 μM), calicheamicin (1 pM) or the combination of calicheamicin (1pM) + M3814 (1 μM). **(C)** Representative images from flow cytometry analysis of apoptosis at 24, 48, 72, and 96 h in MV4-11 cells treated in triplicate with DMSO, M3814 (1 μM), calicheamicin (1 pM) or the combination of calicheamicin (1 pM) and M3814 (1 μM). **(D)** Average percentage of Annexin V-positive cells (early and late apoptotic) from flow cytometry analysis of cells treated in triplicate as in (c) and analyzed at 24, 48, 72, and 96 h. *P < 0.05, **P < 0.01, ***P < 0.001.

Firstly, we examined the cell cycle effect of the combined treatment. MV4-11 cells were exposed to calicheamicin (1.0 pM) and M3814 (1 μM) alone and in combination as in Figure 2 for 24 h and subjected to cell cycle analyses (Figure 3A). M3814 treatment slightly slowed down cell cycle progression as revealed by mild growth delay (Figure 3B). Calicheamicin alone partially arrested MV4-11 cells in G1 phase primarily reducing the S phase population. The combination treatment led to nearly complete cell cycle block in G1 and G2/M phase with only 5% of the cells remaining in S phase, typical for p53-dependent, p21-mediated cell cycle arrest (19, 27) that effectively halted cell growth (Figure 3B). These experiments suggested that the M3814 mediated ATM-dependent p53 boost could contribute to the antitumor effect by strengthening the calicheamicin-induced cell cycle arrest.

Next, we asked if the DNA-PK inhibitor can enhance calicheamicin-induced apoptosis in MV4-11 cells *in vitro*. Again, cells were exposed to calicheamicin (1.0 pM) and M3814 (1 μM) alone and in combination, and the cell population undergoing apoptosis was identified and quantified by staining with phycoerythrin (PE) conjugated Annexin V and 7-amino-actinomycin D (7-AAD) at 24, 48, 72, and 96 h (Figure 3C). The changes in apoptotic cell fraction (combined early and late apoptosis) are summarized in Figure 3D. A time-dependent increase in the fraction of MV4-11 cells undergoing early and late apoptosis was observed in response to calicheamicin alone. The combined treatment resulted in a consistent increase in the fraction of apoptotic cells relative to calicheamicin alone. These results support the hypothesis that by overactivation of the ATM signaling axis M3814 increased calicheamicin-induced p53 transcriptional activity, reinforced p53-dependent cell cycle arrest and apoptosis thus extending the validity of our mechanistic model (16) to acute leukemia cells. In contrast to irradiated solid tumor cells, which acquired p53-dependent premature senescence in the presence of M3814, this boost in p53 activity led to increased calicheamicin-induced apoptosis and cell death in MV4-11 cells. Therefore, M3814-induced p53 overactivation could offer a new approach to enhancing the activity of Mylotarg's warhead calicheamicin in AML cells.

### M3814 Potentiates Calicheamicin Cytotoxicity Independent of p53 in HL-60 Cells

Most AML patients express wild-type p53 in their blasts at diagnosis (22). However, ~10% carry disabling p53 mutants and represent a significant treatment challenge with the established AML therapies (28). The mechanistic model for potentiation of DSB-inducing agents by M3814 in solid tumors cells (16) predicted enhancement of p53-dependent response in p53 wild-type AML cells, but also suggested potentiation of DSB-inducing therapies in p53 dysfunctional cells. These p53-mutant or null cells lack p53-dependent cell cycle arrest and effective protection from mitotic entry with unrepaired DSBs that could lead to mitotic catastrophe (16, 29). In epithelial cancer cells, such outcome was delayed until chromosomal damage accumulates over one or more subsequent cell cycles (16).

**Figure 4:**
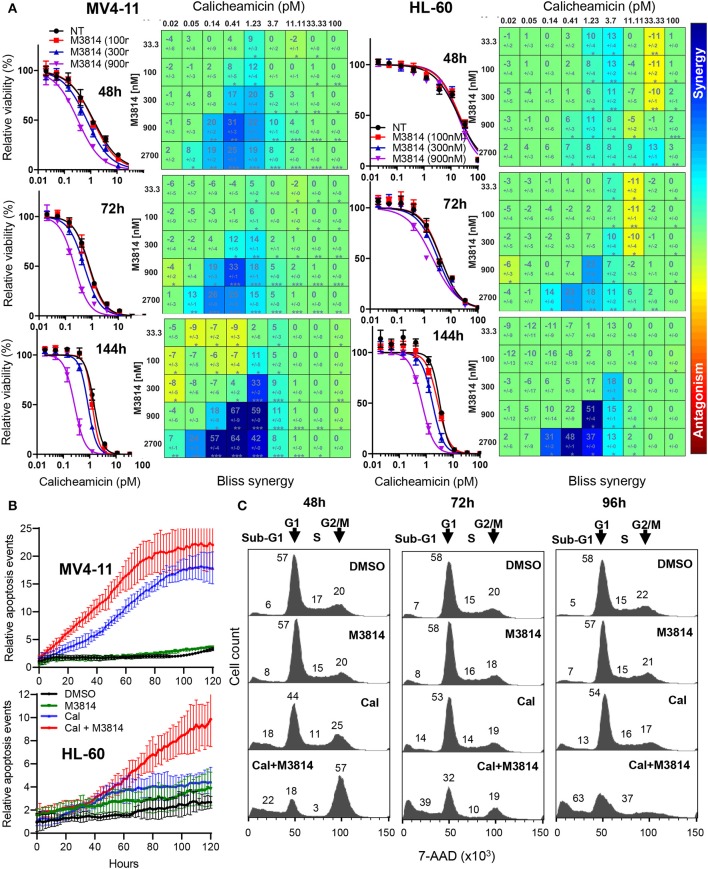
M3814 potentiates calicheamicin cytotoxicity independent of p53 in HL-60 cells. **(A)** Calicheamicin dose-response curves for fixed M3814 concentrations and Bliss synergy matrices for MV4-11 cells (left) and HL-60 cells (right) treated with calicheamicin and M3814 as in Figure 1 and assayed for relative viability using CellTiter-Glo assay at 48, 72, and 144 h. Synergy results were analyzed and graphed using Combenefit software. **(B)** MV4-11 cells (top) and HL-60 cells (bottom) were seeded in 96-well plates and exposed to vehicle (DMSO), M3814 (900 nM), calicheamicin (1.2 pM) or combination of M3814 (900 nM) + calicheamicin (1.2 pM) in the presence of IncuCyte Annexin V reagent and their growth and apoptosis was monitored by live time-lapse imaging by IncuCyte at 10x magnification. Relative apoptosis events were determined from imaging data and calculated as Annexin V-positive events normalized to percent confluence, averaged from eight fields of view across duplicate wells. **(C)** Cell cycle analysis of 7-AAD stained HL-60 cells treated with DMSO, M3814 (1 μM), calicheamicin (5 pM) or the combination of calicheamicin (5 pM) + M3814 (1 μM) for 48, 72, and 96 h. Cells in each phase (sub-G1, G1, S, G2/M) were calculated as a percentage of total cell count (100%) and corresponding numbers positioned in close proximity to the phase they represent.

We examined the mechanism of response to the calicheamicin/M3814 combination in the p53-null AML line HL-60. Firstly, we compared the relative viability of the combined treatment in the p53 wild-type MV4-11 and the p53-deficient HL-60 cell line. Exponentially growing cells were exposed to a combination matrix of a range of M3814 and calicheamicin concentrations, and their relative growth/viability was determined at 48, 72, and 144 h. Dose-response curves generated for three fixed M3814 concentrations (100, 300, and 900 nM) indicated a delayed onset of cytotoxicity for the combination in HL-60 cells compared to MV4-11 (Figure 4A). Our data revealed that M3814 could synergistically enhance the activity of calicheamicin in HL-60 cells but with delayed kinetics.

These findings were supported by live-cell imaging of MV4-11 and HL-60 cells continuously monitoring calicheamicin/M3814 induced changes in cell growth and viability. IncuCyte Annexin V reagent was used to assess real-time apoptosis induction. Annexin V positive cell events were recorded over a period of 5 days and normalized to cell confluence (Figure 4B). Comparison of the curves of relative apoptotic events showed that while M3814 potentiated calicheamicin apoptotic activity in MV4-11 cell continuously, it provided insignificant enhancement in HL-60 cells up to 50 h which was substantially accelerated over the later part of drug exposure. These results are consistent with the hypothesis that p53-dependent apoptosis of AML cells known to occur with rapid onset (22) is the primary driver of the early response to the calicheamicin/M3814 combination in MV4-11 cells. The delayed cell killing of the p53-null HL-60 cells resembled the delayed response of p53 dysfunctional epithelial tumor cells to ionizing radiation and M3814 (16) and suggested that mitotic catastrophe might be involved.

Next, we analyzed the effect of calicheamicin/M3814 treatment on cell cycle progression and viability. HL-60 cells were exposed to calicheamicin (5 pM), M3814 (1 μM) or the combination and their cell cycle distribution was analyzed at 48, 72, and 96 h (Figure 4C). The DMSO control and M3814 treatment exhibited a normal cell cycle profile with traces of apoptotic (sub-G1) population remaining practically unchanged. Cells treated with calicheamicin showed a slight G2/M arrest and increased apoptotic (sub-G1) fraction little changed over the next 2 days. HL-60 cells exposed to the combination were arrested predominantly in G2/M (57%) and had 22% apoptotic (sub-G1) fraction at 48 h. At 72 h, the G2/M peak diminished dramatically giving rise to an increasing (39%) apoptotic (sub-G1) population. At 96 h, most of the cells (63%) were in the sub-G1 population. The remaining 37% of the cell population displayed a profile indicative of ongoing cell death. These results demonstrated that M3814 can substantially enhance the killing potential of calicheamicin in the p53-defficient AML cell line HL-60. Altogether, our experiments revealed that the DNA-PK inhibitor M3814 synergizes with and effectively potentiates the activity of calicheamicin in proliferating AML cells *in vitro*.

### M3814 Shows Strong Combination Benefit With Mylotarg in AML Xenograft Models

**Figure 5:**
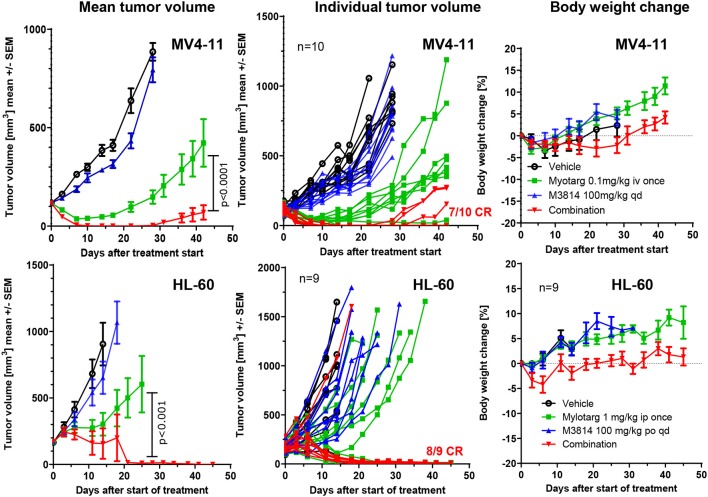
M3814 shows a strong combination benefit with Mylotarg in AML xenograft models. Established subcutaneous MV4-11(group size: 10 mice) and HL-60 (group size: 9 mice) xenograft tumors in immunodeficient mice were treated with Mylotarg (0.1 mg/kg in MV4-11 and 1.0 mg/kg in HL-60, single administration, intravenous injection), M3814 (100 mg/kg, daily, oral gavage), and the combination thereof as indicated. Statistical analysis was performed on log transformed tumor volume data applying a repeated measurement ANOVA with Bonferroni post-test for comparing the groups treated with Mylotarg + M3814 and Mylotarg alone using GraphPad Prism software. CR, complete response.

We sought to determine whether the potentiation of free calicheamicin by M3814 *in vitro* could translate to an *in vivo* combination setting with Mylotarg using mouse xenograft models of AML. MV4-11 and HL-60 tumor xenografts were established subcutaneously in immunodeficient mice and treated intravenously with vehicle, a single dose of Mylotarg (0.1 mg/kg in MV4-11 and 1.0 mg/kg in HL-60) and daily oral doses of M3814 (100 mg/kg), alone and in combination. Animals were monitored over time for tumor volume and body weight changes. M3814 treatment alone did not show a significant effect on MV4-11 tumor volumes as compared to vehicle (Figure 5). Mylotarg treatment led to an initial reduction in tumor volume, and while *3* xenografts had a complete response, tumor outgrowth was observed in *7* animals. Combined treatment with M3814 and Mylotarg, however, resulted in complete responses in 7 of 10 tumor xenografts. Similar results were seen in HL-60 xenografts where no significant activity of M3814 alone on tumor volumes was detected (Figure 5). At the used dose, Mylotarg treatment did not produce a substantial reduction in tumor volume across the treatment group, 3 xenografts had a complete response and tumor outgrowth was observed in *6* animals. Combined treatment with M3814 and Mylotarg, increased the number of complete responses, with no measurable tumor volume in eight of nine treated animals at the termination of the study on day 45. Thus, there was a clear combination effect of M3814 and Mylotarg *in vivo* in both MV4-11 and HL-60 tumor xenografts. Furthermore, this combination had minimal effects on body weight in both MV4-11 and HL-60 studies, indicating potential favorable tolerability (Figure 5).

## Discussion

Using molecular tools and early chemical inhibitors, DNA-PK inhibition has been shown to enhance the antitumor effect of ionizing radiation and DSB-inducing chemotherapeutics and was proposed as a new combination strategy for cancer therapy (30, 31). M3814 is the first potent and selective inhibitor of DNA-PK catalytic activity that has undergone Phase 1 clinical evaluation and is currently being tested in proof-of-concept clinical studies in combination with DSB-inducing therapies. Due to its target selectivity, M3814 offers an excellent molecular probe for mechanistic studies and therapeutic intervention in DSB repair. Recently, we showed that inhibition of radiation-induced DSB-repair by M3814 causes a unique reinforcement of ATM/p53 regulated cell cycle checkpoints and two district cellular responses that are under p53 control: p53 dependent complete proliferation block and premature senescence that protect cells from death or mitotic catastrophe and apoptotic death in the absence of p53 functionality (16). While an enhanced p53 response protects irradiated solid tumor cells from the lethal consequences of radiation-induced DNA damage, the same response in acute leukemia cells may offer a new way for enhanced cell killing via p53-dependent apoptosis. This difference in p53 dependent apoptotic response has been attributed to the fact that that majority of solid tumors expressing wild-type p53 acquire defects in the p53-dependent apoptotic signaling (19, 32) while most acute leukemias are known to preserve p53 wild-type status and apoptotic function (22).

Here, we investigate the applicability of this approach to combination therapy of DNA-PK inhibitor M3814 with Mylotarg, an ADC armed with a potent DSB-inducing warhead, calicheamicin. Our results demonstrate that calicheamicin activity is synergistically potentiated by M3814 in p53 wild-type MV4-11 and MOLM-13 cells *in vitro*. The significant overactivation of the p53 pathway and induction of cell cycle arrest and apoptosis in MV4-11 cells suggests that p53-dependent cell cycle arrest and apoptosis is an important contributor to the potentiation of calicheamicin in the p53 wild-type setting. Our results do not exclude p53-independent killing of p53 wild-type AML cells. Indeed, it has been shown that calicheamicin can induce apoptosis in p53-null clone of the p53 wild-type HCT116 cancer cell line but did not investigate the possibility for involvement of different mechanisms (11). Engaging the p53-dependent apoptotic signaling has been established as an important component of current AML therapies with proven clinical success and its synergistic enhancement by a DNA-PK inhibitor offers a new approach for potential therapeutic intervention.

A different mechanism is likely behind the enhanced activity of calicheamicin/M3814 combination in the p53 null HL-60 cell line. In the absence of functional p53, a weakened checkpoint control allows a larger number of cells to enter mitosis with unrepaired DSBs, frequently leading to mitotic catastrophe (29). The slower onset of cell death in HL-60 cells during calicheamicin/M3814 treatment and the predominant G2/M arrest giving rise to an increasing apoptotic (sub-G1) population remarkably resembling the fate of irradiated HeLa cell under M3814 treatment (16) hint to involvement of mitotic catastrophe (33). However, apoptotic or necrotic cell death independent of mitotic catastrophe in p53-deficient cells cannot be excluded as a contributor to the overall enhanced AML cell killing in response to the calicheamicin/M3814 combination. Future focused studies in panels of p53-deficient cell lines are needed to establish the predominant mechanism of M3814 enhanced calicheamicin-induced cell death in AML.

The results described in this manuscript suggest that regardless of the p53 status of AML cells and the mechanisms of response, DNA-PK inhibition effectively sensitizes AML cells to Mylotarg *in vitro* and *in vivo*. Thus, M3814 could offer a new combination approach to AML therapy with a potentially improved treatment outcome. Selectively targeting the CD33-positive AML cells may spare normal bone marrow cells from enhanced p53-dependent toxicity. Indeed, our mouse models revealed a minimal increase of body weight loss which was fully reversible. However, the true safety window of such a combination strategy could be determined only in the proper clinical setting.

Antibody drug conjugates with several deferent DNA damaging payloads have been reported and are at different stages of preclinical and clinical evaluation (34). They may offer interesting new opportunities for combination with inhibitors of DNA repair pathways, such as M3814.

## Data Availability Statement

The datasets generated for this study are available on request to the corresponding author.

## Ethics Statement

The animal study was reviewed and approved by Animal Welfare Committee, Merck KGaA, Darmstadt, Germany.

## Author Contributions

MC, AZ, L-YC, and LV designed the experiments. MC, AZ, and L-YC performed experiments, analyzed and graphed data. LV, MC, and AZ wrote the manuscript. LV, AZ, MC, FZ, and AB discussed experimental data, revised and edited the full content of the manuscript. All authors have read, revised critically and approved the final version of the manuscript.

### Conflict of Interest

All authors are employees of Merck KGaA or its subsidiary EMD Serono, Inc.
